# Pulmonary Hernia in a Two-Year-Old Child

**DOI:** 10.1155/2014/792376

**Published:** 2014-09-28

**Authors:** Jenna Fine, Bryan S. Walters, Alysia A. Agnoni, Christopher P. Coppola, Ronald J. Scorpio, Alfred P. Kennedy

**Affiliations:** Department of Pediatric Surgery, Geisinger Health System, Janet Weis Children's Hospital, 100 N. Academy Avenue, M.C. 21-70, Danville, PA 17822, USA

## Abstract

Pulmonary hernia, also known as lung herniation or intercostal herniation, is best explained as the lung parenchyma protruding beyond the confines of the thoracic wall. This rare finding can be classified as congenital or acquired. Acquired pulmonary herniations are often the complication of blunt or penetrating trauma to the chest wall. This report describes a two-year-old male who fell onto a rigid post, striking his left lower chest. Imaging studies demonstrated a small pneumothorax as well as pulmonary herniation. The patient underwent a diagnostic thoracoscopy and repair of a pulmonary hernia within the 7th intercostal space without complication. In this case report, we aim to add to the limited body of existing literature on the surgical management of pulmonary hernias.

## 1. Introduction

Herniation of the lung is defined as the protrusion of pulmonary tissues through the confines of the thoracic cage [1]. In the pediatric population, most lung herniations are the direct result of the disruption of the intercostal muscles and fascia secondary to the laxity of the pediatric thoracic cage [1]. The therapeutic approach may include observation or surgical repair, with or without mesh. We present a case of a two-year-old child who suffered a blunt injury to the chest wall, which resulted in a pulmonary hernia and surgical repair.

## 2. Case Report

**Figure 1:**
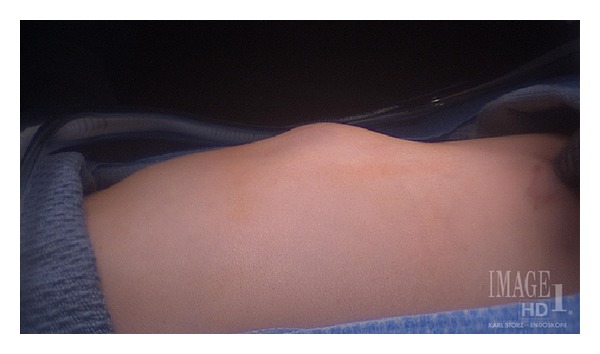
View of the lateral chest wall demonstrates a protrusion in the region of the left lateral 7th rib. The patient is lying in the right lateral recumbent position.

A two-year-old male fell onto a rigid post approximately one centimeter in diameter while playing. Physical examination of the left lateral inferior chest wall revealed a mass with paradoxical motion during respiration (Figure 1). There was an area of ecchymosis noted in the left lower rib area.

**Figure 2:**
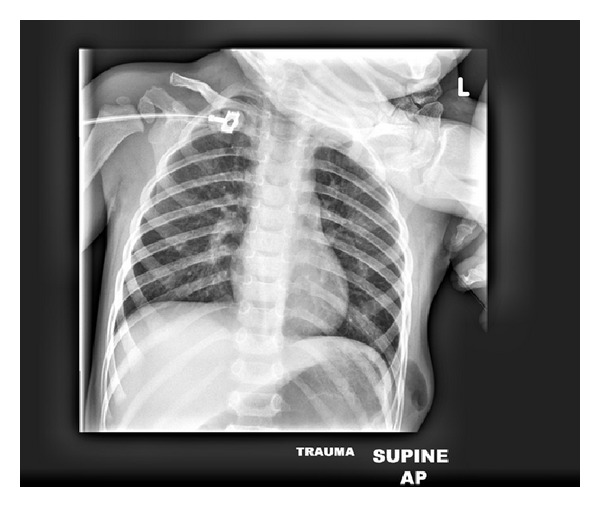
Chest radiograph reveals a tubular soft tissue lucency.

Radiographs of the chest demonstrated a tubular lucency within the soft tissues of the 7th intercostal space. No pneumothorax or rib fractures were evident (Figure 2). Computed tomography (CT) of the chest was utilized as part of the overall trauma evaluation. Chest CT showed a small pneumothorax with left lower-lobe pneumatoceles. In addition, a small amount of air was noted adjacent to the ribs and within the muscle at the site of injury, raising the concern for a traumatic pulmonary hernia.

**Figure 3:**
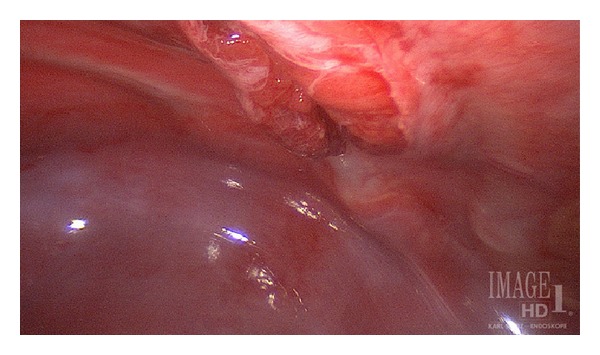
Thoracoscopic image of the pulmonary hernia at the lateral 7th intercostal space.

The patient underwent diagnostic thoracoscopy. Findings included an intact left hemidiaphragm as well as a small area of contusion to the lingular portion of the left lung. Associated with this was a disruption of the pleural lining and the overlying musculature within the 7th intercostal space (Figure 3). Surgical repair of the defect was performed. Full disruption of the musculature was noted with communication into the pleural space. Primary repair was conducted with suture closure of the muscular fascia without the use of synthetic graft or pericostal sutures. At the end of the operation, a 16-French thoracostomy tube was inserted to drain the pleural space. The child recovered without untoward event.

The patient was seen in follow-up seventeen months following repair. The parent reported good health since surgery, and the wound was well-healed. There was no evidence of recurrent hernia.

## 3. Discussion

Intercostal hernias, otherwise known as pulmonary hernias, are a protrusion of body cavity contents through adjacent ribs due to a defect located in the musculature [2]. Pulmonary hernias can be classified as congenital or acquired [3]. Congenital hernias are the result of a weakness in the thoracic wall or an increased intrathoracic pressure. Acquired hernias are further classified into traumatic or pathological hernias, wherein weakening of the thoracic cage is caused by injury or disease, respectively [4].

Acquired hernias are usually associated with some type of blunt trauma to the chest wall. Blunt trauma resulting in herniation of the lung is extremely rare in the pediatric population. It is postulated that the elastic nature of a child's thoracic wall absorbs enough of the kinetic energy to prevent such herniation [4]. It has been noted that the object which is causing the blunt trauma must be smaller than the width of an intercostal space. Furthermore, the object must strike within the space, not the rib. Although not applicable to our patient, these herniations are often associated with rib fractures [5]. Penetrating injury may also result in herniations but is far less common [6]. The most common source of pulmonary hernia remains motor vehicle accidents [7].

On physical exam, symptoms can be very subtle or can speak for themselves. Since this is so rare, the diagnosis is often missed. If an intercostal mass is present, it can be expected to vary in size with respiration and to have paradoxical protrusion during expiration. The intercostal mass will be reducible and elastic and may be associated with an overlying crepitus [1]. On chest radiographs, the most common finding is a well-circumscribed loculation of subcutaneous air. Whether or not the herniation is visible on chest radiograph, a CT should be performed to better visualize the thoracic cage and pleural space and to rule out associated injuries [7].

Treatment options include observation or surgical repair. Controversy exists regarding which treatment option should be employed. Factors associated with repair include the location of the hernia and the presence of symptoms. Due to the small size of the defect, our patient was treated with suturing of the muscular fascia. Larger defects, however, may require repair with mesh. Some authors have reported that supraclavicular herniations may be observed, while intercostal herniations require immediate repair [8]. It has also been stated that asymptomatic cases may be observed, while those with hemoptysis, recurrent respiratory infections, or those threatening to strangulate, must be repaired [4].

In our case, we elected to proceed with surgical repair of the pulmonary hernia due to risks of future strangulation and the noted size and site of the defect. Though there is a limited body of literature on pulmonary hernia, it is reasonable to argue that entrapment of herniated lung may lead to ischemic complications of lung tissue [9]. We also feel that should a repeat injury to the same region occur then there is conceivably an increased risk of intrathoracic injury. Our patient tolerated the procedure well and had no complications. We suggest diagnostic thoracoscopy be considered in any child who presents with a possibility of pulmonary herniation to further quantify the injury to the chest wall as well as other potential injuries within the thorax. Thoracoscopy allowed for superb visualization of the intrathoracic wall and lung parenchyma, and the injury was easily accessible for repair. It is our thought that the potential complications of not repairing the hernia are greater than those associated with operative repair.
